# High-power and high-beam-quality with unstable resonator in a Yb:YAG slab laser

**DOI:** 10.1371/journal.pone.0310000

**Published:** 2024-12-05

**Authors:** Jian Lei, Wei Jiang, Ruihan Dong, Gaozinan Yang, Jie Li, Xueshuang Deng

**Affiliations:** 1 Electronic Information and Electrical College of Engineering, ShangLuo University, Shangluo, Shaanxi, China; 2 Institute of Quantum Optics and Quantum Information, ShangLuo University, Shangluo, Shaanxi, China; Universiti Brunei Darussalam, BRUNEI DARUSSALAM

## Abstract

The development and implementation of an unstable resonator laser system were explored in the present study, employing dual-end pumping of a Yb:YAG slab laser via laser diode (LD) arrays. The proposed system exhibited significant improvements in both power output and beam quality. The experimental design and execution of the laser oscillator were conducted under controlled conductive cooling conditions. Operating at a current of 90 A, the system achieved a total output power of 3823 W, with continuous-wave laser emission at 1030 nm observed bidirectionally. The measured beam qualities were 1.737 and 2.085, indicating that the system maintained good beam quality while achieving high power output. Additionally, an investigation into the optical-to-optical conversion efficiency was conducted, yielding a value of 35%. The findings underscore the exceptional performance of the devised unstable resonator within Yb:YAG slab lasers, with anticipated pivotal contributions to the broader laser domain. Novel approaches towards attaining high power output and superior beam quality laser sources were introduced, marking substantial advancements in Yb:YAG slab laser technology. The implications of these findings extend beyond the realm of laser technology, providing valuable insights applicable to a diverse range of optical, thus allowing the study’s impact to transcend its immediate field.

## 1. Introduction

High-power lasers exhibiting superior beam quality are essential across diverse sectors, particularly in materials processing applications [1–3], industrial technologies [4,5], and scientific research [6,7]. The demand for such lasers has driven the development of advanced laser systems capable of delivering high levels of power and beam quality. Within the domain of solid-state lasers, the high-power slab laser has emerged as a critical component, significantly contributing to the field’s portfolio of key technologies [8,9]. The Yb:YAG slab laser configuration has attracted significant research interest due to its advantageous thermal and optical characteristics, positioning it as a promising candidate for high-power laser applications [10]. Nevertheless, conventional LD-pumped solid-state lasers face significant constraints on power output and beam quality due to thermal effects. To address such challenges, extensive research has been conducted, resulting in the proposal of practical solutions. Notably, the utilization of surface gain slab laser systems has emerged as a promising approach to mitigate thermal effects in solid-state lasers [11,12]. Oscillators have seen diminished favor in achieving high power within slab lasers, largely due to the notable length-to-width ratio of their rectangular apertures. This geometric constraint has led to a preference for amplification methodologies in high-power slab laser systems. Multi-mode stable resonators are frequently employed in the assessment of slab amplifiers [13]. Yet, the occurrence of multi-mode output presents a significant drawback for high-power laser applications due to the resultant deterioration in beam quality. Moreover, traditional stable resonator configurations demonstrate limitations in terms of weak characteristic extraction, susceptibility to multiscale noise interference, and the imposition of fixed rescaling factors, thus restricting their potential for improvement. As such, research efforts have been directed towards exploring alternative methodologies for power extraction from slab lasers, with particular emphasis on the investigation of diverse unstable oscillator configurations [14–17]. N. Hodgson, et al. developed unstable resonator configurations for both rod and slab lasers, demonstrating the potential for high output power while maintaining superior beam quality [18]. X. Wang et al. documented the realization of a 1350W Nd:YAG slab laser employing an unstable ring resonator configuration with effective reverse wave suppression, thereby achieving the highest recorded output power from an unstable ring laser with proficient reverse wave suppression [19]. D. Mudge et al. devised a novel approach for constructing a high-power, continuous-wave Nd:YAG laser characterized by diffraction-limited performance. Their innovative design integrates side-pumped, side-cooled zigzag slabs within a hybrid stable-unstable resonator configuration. Significantly, this architecture can achieve a geometric magnification factor of at least 1.3 in the unstable axis [20]. In 2012, Pargmann C et al. reported an unstable resonator laser with an output power of 2 kW, M^2^ of 3.4 [21]. So this is the highest level we know of to date.

The primary challenge in implementing slab geometries within an unstable resonator configuration stems from their substantial thickness, typically on the order of several millimeters. Consequently, the insufficient Fresnel number along the thickness dimension inhibits the achievement of unstable output. To address the conflicting requirements of high performance—specifically in terms of power output and beam quality—and practical applicability, such as compactness and robustness, this investigation focuses on the design and experimental validation of a hybrid resonator for a 3.8 kW-class high-power slab laser. The proposed resonator architecture incorporates both flat-flat and unstable resonator elements, oriented orthogonally with respect to the rectangular aperture of the slab. Specifically, the flat-flat resonator component aligns with the thickness dimension of the slab, while the unstable resonator element extends along its width. This design optimizes mode quality and output beam efficiency, leveraging fundamental mode operation along the thickness direction and unstable mode operation along the width direction. Hence, the resultant laser system exhibits a compact and streamlined structure reminiscent of an oscillator. Notably, the laser demonstrates an output power of 3823 W corresponding to a pump power of 10904 W (90A). The optical-to-optical conversion efficiency is reported at 35%, with a beam quality factor of $M_{x}^{2}=1.737,M_{y}^{2}=2.085$. To the present knowledge, this represents the highest reported output power and beam quality for a large-scale slab laser oscillator configuration.

The innovation highlighted in the present study emphasizes the integration of dual-end pumping using an LD array with a Yb crystal and the implementation of an unstable resonator slab laser configuration. This configuration incorporates an elongated Conduction-Cooled End-Pumped Slab (CCEPS) structure’s surface gain slab within an off-axis confocal hybrid resonator, analogous to a Keplerian telescope. This arrangement enables the generation of kilowatt-level continuous laser output with both high power and high beam quality.

## 2. Experimental setup

In the present study, a non-stabilized, positively branched off-axis virtual confocal structure of the telescope system was chosen. This configuration is capable of producing high-quality laser beams with reduced losses compared to conventional unstable resonators. A column lens design was employed, resulting in a hard edge output. Additionally, the geometrical length of the unstable cavity was adjusted by taking into account the insertion of optical elements within the resonator.

**Fig 1:**
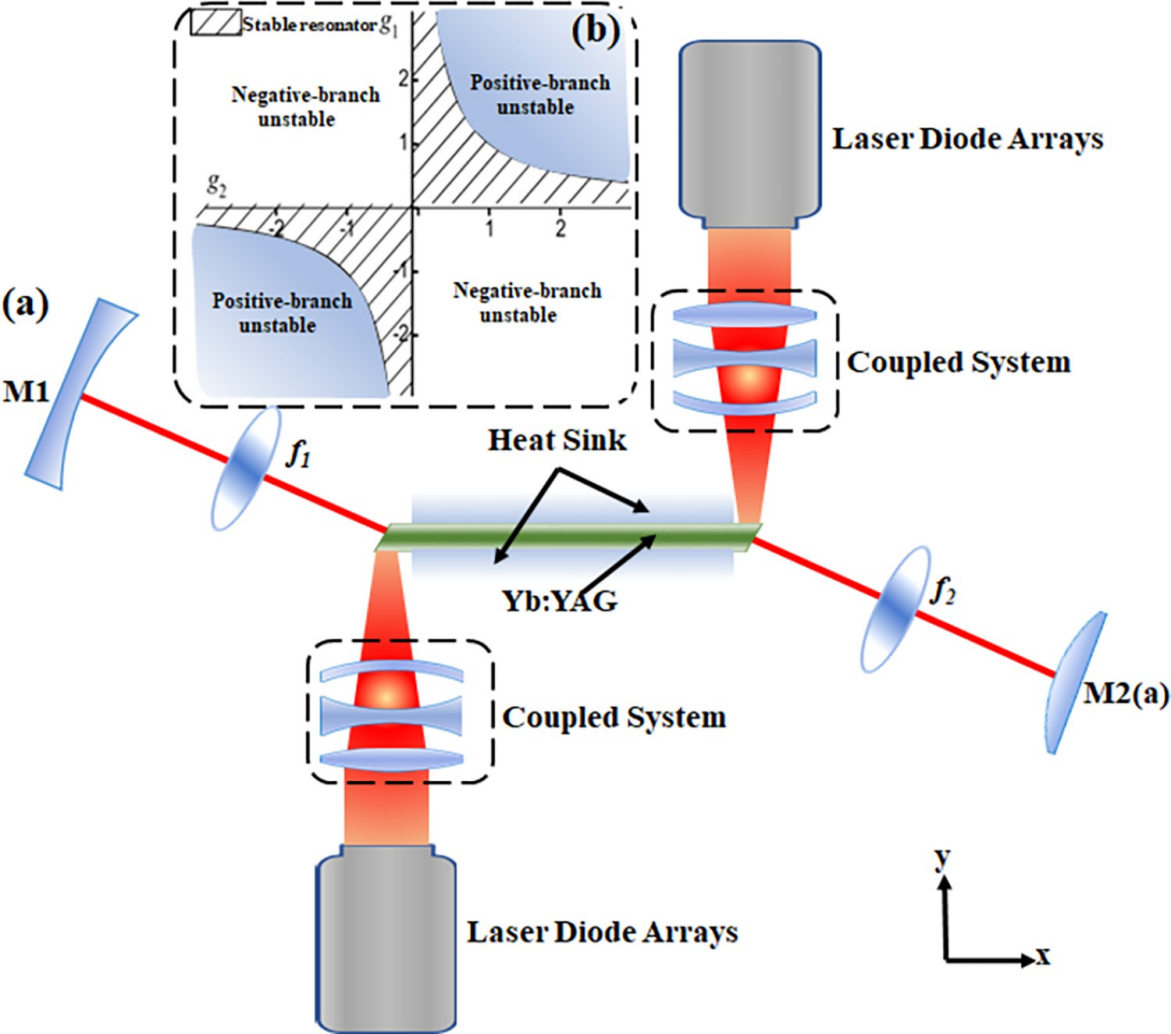
(a) Schematic of the experimental setup, (b) Stability Diagram of all laser resonators. (a) the schematic diagram of the experimental setup for the laser diode (LD) array dual-end pumped Yb:YAG slab laser, which emitted at a wavelength of 1030 nm. (b) shows a plot of the stability interval distribution of a resonator, with the positively branched and negatively branched unstable regions labeled, and the shaded region indicating the stable resonator region.

The distribution of positive and negative branching confocal unstable resonator is shown in Fig 1(B). The geometric magnification is determined by the curvature ratio of M1 and M2(a). The distance L between the lenses is given by f^1^ plus f^2^, where f^1^ and f^2^ are the focal lengths of lenses M1 and M2(a), respectively. The most obvious difference between such resonator is that the negatively branched unstable resonator has an intracavity focus. In high-power systems, intracavity focusing can lead to airborne faults. When the length of the gain medium constitutes a significant fraction of the total resonator length L, a positive-branch resonator is typically favored due to its enhanced gain extraction efficiency.

Fig 1(B) shows a plot of the stability interval distribution of a resonator, with the positively branched and negatively branched unstable regions labeled, and the shaded region indicating the stable resonator region. The g-parameter plots show the stable region of the cavity (shaded portion), and therefore also indicate the unstable region of the cavity (unshaded). The g-parameter is defined by Eq (1):

$$g_{i}=1-\frac{L}{R_{i}},i=1,2$$ (1)

where and are the radius of curvature of mirrors M1 and M2(a). The resonator is stable if 0<*g*_1_*g*_2_<1. If the g-parameter falls outside this range, the resonator is unstable.

1. Determination of magnification M
$$T_{out}=(\sqrt{2g_{o}l/L}-1)L$$ (2)
  where *g*_*o*_ is the small signal gain, *l* is the length of the gain medium, and *L* is the total loss.
  The relationship between the optimum magnification M and the optimum *T*_*out*_ transmittance of the unstable resonator is shown in Eq (3):
$$T_{out}=1-\frac{1}{M}$$ (3)
  Substituting specific values, the following can be obtained: *T*_*out*_ = 0.6、*M* = 2.5.
2. Determination of output coupling mirror M2(a) size
  To achieve effective mode discrimination in an unstable resonator, an appropriate resonator length is selected such that the equivalent Fresnel number is a half-integer. The relationship between the equivalent Fresnel number, the cavity length, and the size of the output coupling mirror M2(a) is described by Eq (4):
$$N_{eq}=\frac{(M-1){\left(a\right)}^{2}}{2L\lambda }$$ (4)
  Considering the specific experimental conditions, the resonator length is selected *L* = 1000*mm*. From the width-direction dimension of the gain medium *w*≥*Ma*, the size of the output coupling mirror M2(a) can thus be determined *a*, *a*≤11.2*mm*. The size of the output coupled mirror M2(a) is *a* = 11.2*mm* when the equivalent Fresnel number *N*_*eq*_ = 84.5.
3. Determination of resonator mirror curvature *R*_1_ and *R*_2_
  The curvature of the resonator mirror is shown in Eq (5):
$$R_{1}=2ML/M-1,\;R_{2}=-2L/M-1$$ (5)
  so, *R*_1_ = 3333*mm*, *R*_2_ = −1333*mm*, $g_{1}=1-\frac{L}{R_{1}}=0.7$, $g_{2}=1-\frac{L}{R_{2}}=1.75$
  *g*_1_*g*_2_ = 1.23>1, that is, a positively branching unsteady cavity in the slat width direction, which satisfies the design requirements.
  In this resonator, the presence of an optical gain medium causes the focal point of the reflective surfaces to shift due to refraction effects. To maintain confocal alignment, it is necessary to correct the resonator length to compensate for this drift. When there is a gain medium with a refractive index of (Yb:YAG refractive index *n* = 1.82, *λ* = 1030*nm*) and a resonator length of *l* = 121*mm*, the focal point offset Δ*L* is corrected by the resonator length:
ΔL=l(1−1/n)=54.4mm
  Then, the actual cavity length of the resonant cavity is:
L'=L+ΔL=1054.4mm

Fig 1(A) depicts the schematic diagram of the experimental setup for the laser diode (LD) array dual-end pumped Yb:YAG slab laser, which emitted at a wavelength of 1030 nm. The pump source comprised a 28-bar microchannel water-cooled continuous output semiconductor laser array, with each bar equipped with a micro-lens to collimate the pump light in the fast axis. The pump light coupling system featured two sets of cylindrical lenses, achieving an efficiency of approximately 92%. This system shaped the pump light into a uniform distribution along the x-axis and a Gaussian or super-Gaussian distribution along the y-axis, with the pump area dimensions being approximately 2 mm × 28 mm. The doped area dimensions were measured at 100 mm × 28 mm × 0.3 mm, as depicted in Fig 1, with a Yb^+3^ ion doping concentration of 0.35 at.%. The slab was cut at a 45° angle, and its end faces were coated with anti-reflection (AR) coatings at wavelengths of 940 nm and 1050 nm. Moreover, the sides of the slab underwent edging and sanding processes to mitigate amplified spontaneous emission (ASE) and suppress parasitic oscillations. The two 121mm×28mm faces are the cooling surfaces. These surfaces are cooled using a copper heat sink equipped with microchannel water cooling. To ensure uniform heat distribution and enhance heat conduction, metal indium is vacuum brazed between the crystal and the heat sink.

As depicted in Fig 1(A), the resonator configuration incorporated cavity mirrors M1 and M2(a). M1 featured a flat-concave cylindrical reflective mirror with a curvature radius of R = 3333mm, coated with a high-reflection film tailored specifically for 1030nm wavelength. In contrast, M2(a) functioned as the flat-convex cylindrical mirror with a curvature radius of R = -1333 mm, serving as the laser resonator’s output mirror. Further, lenses f1 and f2, each with a focal length of 300 mm, were integrated into a thermally stable 4f system. This carefully designed setup effectively mitigated the adverse effects of thermal fluctuations on the output characteristics and significantly improved coupling efficiency. The packaging method of this slab laser crystal is as follows: First, the surface of the slab laser crystal is coated with an optical film (Silicon dioxide film with a thickness of 2.5um, a highly reflective film) and a metal film in turn(a titanium-platinum film, of which the thickness of titanium film is 150nm, the thickness of platinum film is 100nm, and the thickness of gold film is 600nm.), and a gold film (with a thickness of 500nm) and an indium layer(with a thickness of 80nm) are coated on one side of the heat sink in turn. Next, the upper and lower surfaces of the coated slab laser crystal are connected to the heat sink with the indium layer to form a welded body. Finally, in a vacuum welding furnace, pump light is injected from a non-welded port of the welding body, and the slab laser crystal is heated and packaged by utilizing the heat generated by the absorption of the pump light by the slab laser crystal. This design choice reduced thermal resistance, thereby enhancing the slab’s heat dissipation capabilities. As such, the configuration enabled efficient propagation of high-power pump light and precise laser injection, leading to a substantial increase in laser output power. In parallel, the doped slab’s surface layer played a crucial role in preserving the laser’s intricate "zigzag" optical path. This meticulous attention to detail served to minimize wavefront distortion, thereby elevating the overall quality of the output laser beam.

The detailed output characteristics of the Yb:YAG slab laser at a wavelength of 1030 nm, under the operational mode of the laser resonator, are comprehensively outlined in Table 1.

**Table 1:** Parameters for the calculation of the Yb:YAG slab laser and structural details of the mixing resonator configuration.

Yb:YAG Slab Size	121mm(L)*28mm(W)*2mm(T)
Slab construction type	double surface gain slab structure
Doped area size	100mm(L)*28mm(W)*0.3mm(T)
Doped concentration	0.35%at
Refractive index	1.82
Planck constant	6.626*10^-34^J.s
Speed of light	2.998*10^8^m.s^-1^
Boltzmann constant	1.381*10^-23^J.K^-1^
Pump optical power	12kW
Cooled temperature	288K
Pump light wavelength	940nm
Laser wavelength	1030nm
Pump light absorption cross section	7.6*10^-21^cm^2^
Stimulated emission cross section	0.3*10^-20^cm^2^
Fluorescence lifetime	951μs
Thermal conductivity	0.11W.cm^-1^K^-1^
equivalent Fresnel number	84.5
the laser resonator’s output mirror M2 (a)	11.2mm
Optimum magnification M	2.5
Optimal transmittance	0.6

**Fig 2:**
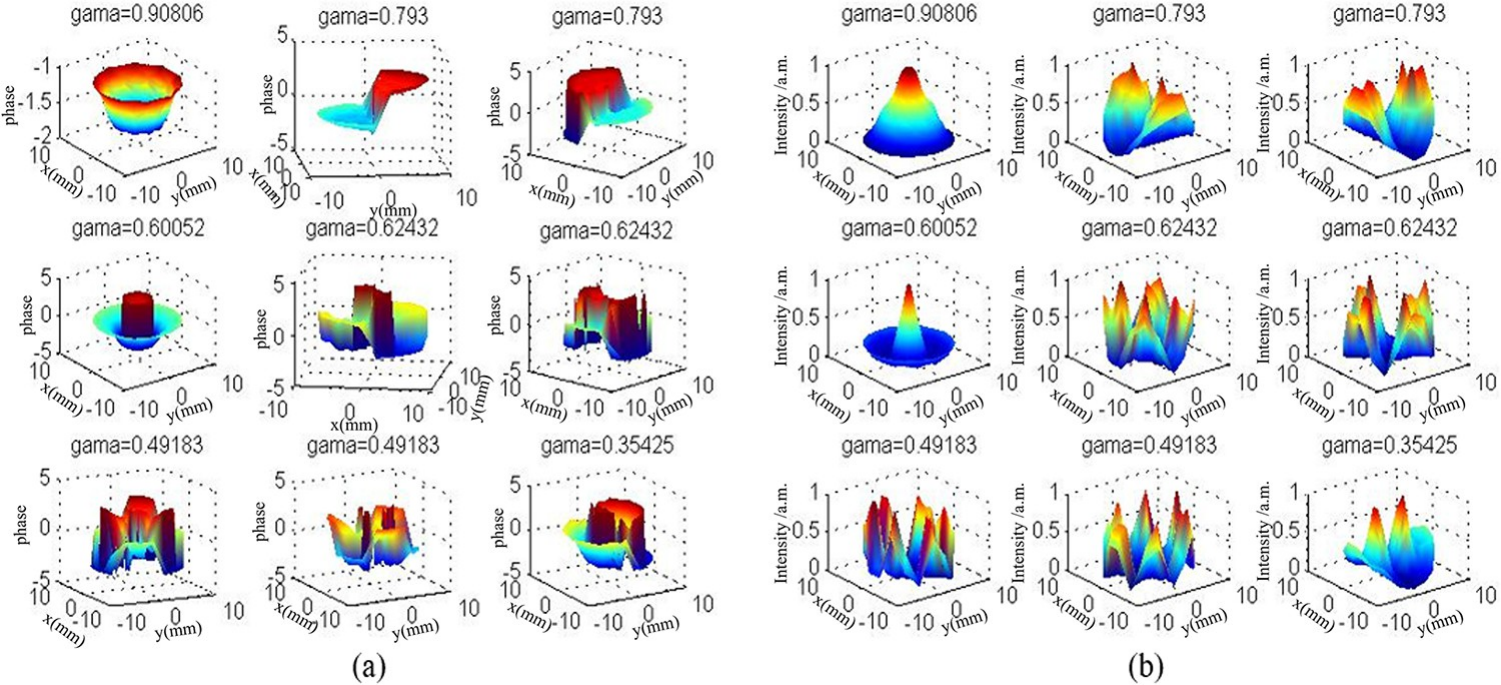
Near-field phase distribution(a), light intensity distribution(b) in unstable resonator mode. The gama is an eigenvalue of the eigenvector method of simulation for calculating the oscillatory modes in an unstable resonator. The x and y axes represent the size of each mode scale, and the z axis represents the phase (Fig 2(a)) and the normalized light intensity (Fig 2(b)).

From Fig 2, an observation can be made that a larger fundamental mode volume could be obtained by using the unstable resonator structure. At the same time, the loss difference of each mode was larger, and the mode identification ability in the resonator was stronger. Therefore, the unstable resonator was conducive to obtaining high power and high beam quality simultaneously. And the gama is an eigenvalue of the eigenvector method of simulation for calculating the oscillatory modes in an unstable resonator. It describes the unidirectional phase shift and unidirectional loss of the corresponding mode in the resonator. The main advantage of the eigenvector method over the conventional method is that a series of mode distributions can be obtained simultaneously in a single computation, and the mode recognition capability of the resonator can be analyzed directly on the basis of the losses in each module. The x and y axes represent the size of each mode scale, and the z axis represents the phase (Fig 2(A)) and the normalized light intensity (Fig 2(B)).

## 3. Experimental results

The unstable resonator addresses the limitations of a stable resonator and offers two significant advantages: a larger mode volume, which enhances the utilization of the gain medium, and improved mode discrimination capabilities, facilitating single-mode operation. Specifically, while the unstable cavity excels in mode selection, it also provides a substantial fundamental mode volume, which is beneficial for achieving high-power, high-beam-quality laser output. The mode distribution on the output coupler of the square mirror unstable cavity is calculated using the characteristic vector algorithm of the transfer matrix.

Using an unstable cavity structure can achieve a larger fundamental mode volume, while the losses of each mode differ significantly, indicating strong mode discrimination capability within the resonator. Thus, an unstable cavity is advantageous for achieving both high power and high beam quality.

**Fig 3:**
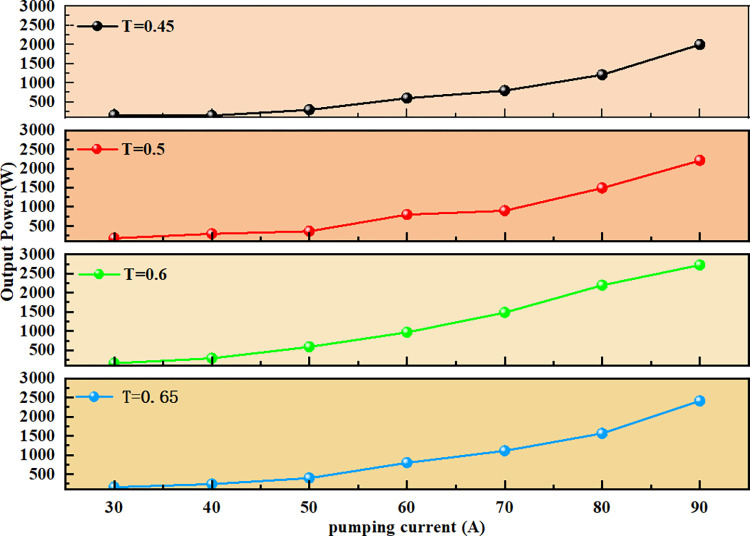
Laser output power curves for the unstable resonator at different transmittances. an observation can be made that the optical-to-optical conversion efficiency was highest when the transmittance was 60%, making it the most suitable for the operation of this laser system.

In order to obtain the optimal coupling output efficiency of the resonator used in the present experiment, = a 1030mm HR plane mirror was used as the rear mirror of the cavity. Concave mirrors with transmittances of 45%, 50%, 60%, and 65% were then used as the output mirrors to form a plane-concave cavity and test the efficiency of the laser oscillator. By adjusting the resonator, the maximum laser output was achieved. Laser output power curves were generated for different transmittances of the output mirror, as shown in Fig 3.

**Fig 4:**
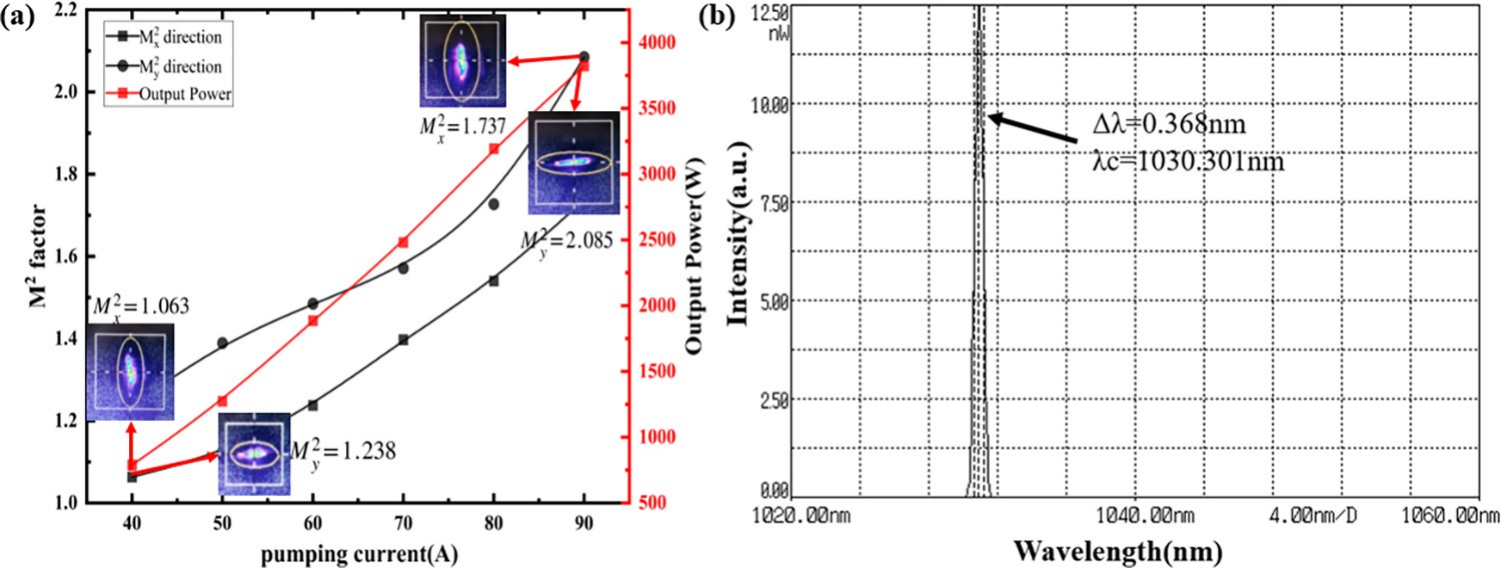
(a) Pump current in direct relation to M^2^ factor and output power; (b) the output spectrum at 1030nm. (a) the pump current was set to 40A, 50A, 60A, 70A, 80A, and 90A, M^2^ beam analyzer measurements were conducted to assess the M^2^ factor curves and near-field spot diagrams in both the horizontal (x direction) and vertical (y direction) directions. (b) the spectral widths at 1030nm.

Using the experimental setup described, a stable continuous laser output at 1030nm was successfully achieved. With a pumping current of 90A, a laser output of 3823W was attained, resulting in an optical-to-optical conversion efficiency of 35%. The pump current in direct relation to M^2^ factor and output power is depicted in Fig 4(A). Notably, as the pump power at both ends of the laser increased to 2000W, the laser output power exhibited linear growth with the pump power, without any indication of the curve flattening. Such findings suggest the absence of gain saturation within the laser resonator during this period. For this experiment, a resonator with an amplification factor (M) of 2.5 was selected. Additionally, when the pump current was set to 40A, 50A, 60A, 70A, 80A, and 90A, M^2^ beam analyzer measurements were conducted to assess the M^2^ factor curves and near-field spot diagrams in both the horizontal (x direction) and vertical (y direction) directions, as illustrated in Fig 4(A). The laser output spectrum of 1030nm laser with an absorbed pump power of 10904W (pumping current of 90A) is shown in Fig 4(B). In the experiment, the HighFinesse Spectrometers · LSA · 1–2022 was used for testing. The spectral widths were 0.368 nm centered at 1030.301 nm.

**Fig 5:**
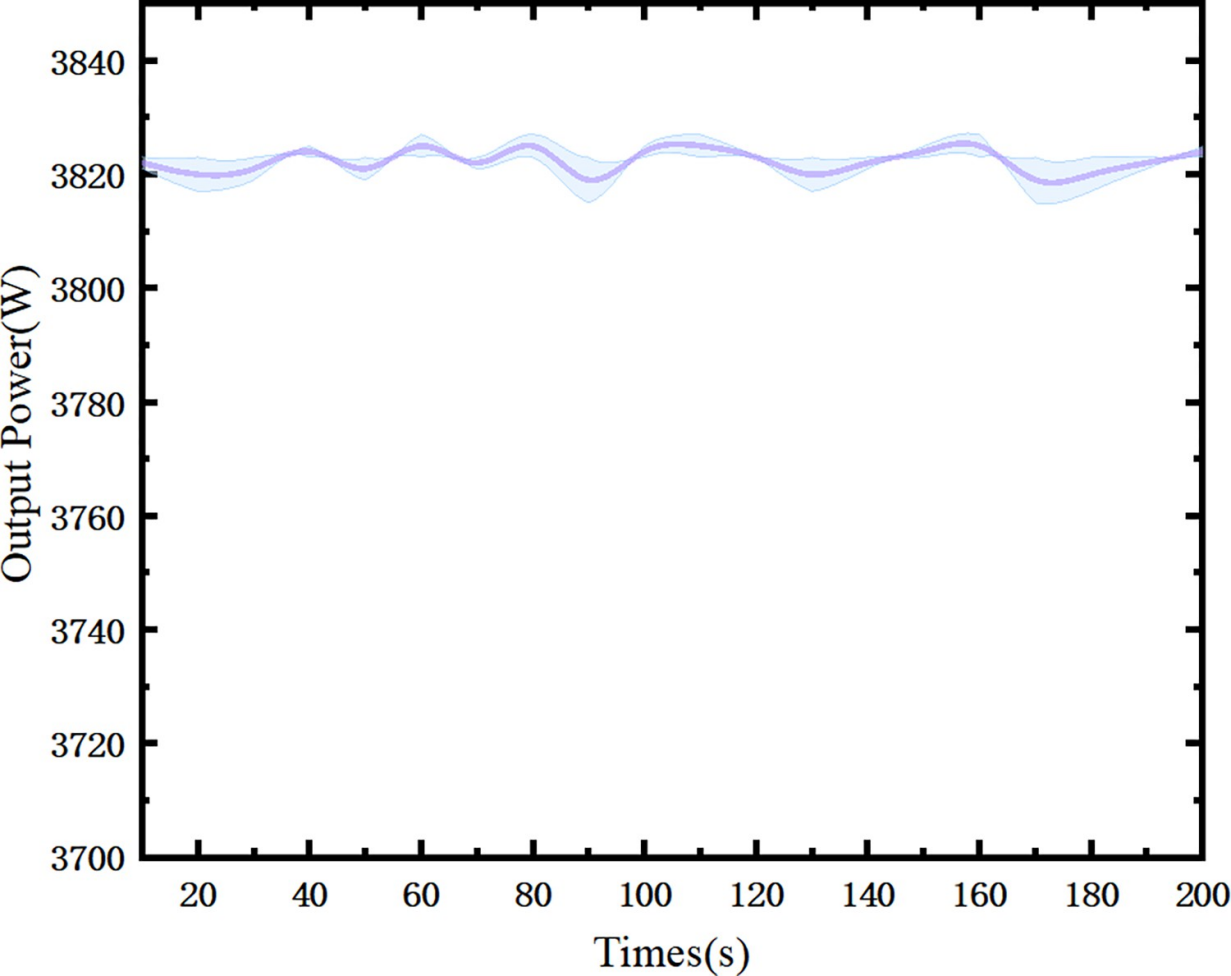
Time stability of the output power at 3823W. Fluctuations in the output power within 200 seconds, indicating a time stability of less than 1.26%.

Additionally, measurements were taken every 10 seconds for the output power at 3823W, and the results are shown in Fig 5. An observation can be made that there were no significant fluctuations in the output power within 200 seconds, indicating a time stability of less than 1.26%.

## 4. Conclusion

In conclusion, a novel resonator laser utilizing LD array dual-end pumped Yb:YAG slab laser technology was established. The proposed system can achieve high power and high beam quality. The design and experimentation under conductive cooling conditions demonstrated the effectiveness of this laser oscillator. At a current of 90A, the system achieved a notable output power of 3823W with continuous laser output at 1030nm in both directions, maintaining good beam quality with measured values of 1.737 and 2.085. Additionally, the optical-to-optical conversion efficiency was found to be 35%. These findings highlight the exceptional performance of the designed unstable resonator within Yb:YAG slab lasers, showcasing its potential significance in the broader laser technology landscape. Further, the present study provides valuable insights into achieving high power and high beam quality in laser sources, marking significant advancements in Yb:YAG slab lasers with implications beyond laser technology, extending to various optical applications.

## Supporting information

S1 Raw data These excel spreadsheets contain the raw data from the experiments that were analyzed in this article. It is separated by subject as well as by measurement type and measurement number. The caption for the Supporting Information files is “Raw data”.
(ZIP)
